# The dualism between adatom- and vacancy-based single crystal growth models

**DOI:** 10.1038/s41467-019-13188-0

**Published:** 2019-11-20

**Authors:** Marcel J. Rost, Leon Jacobse, Marc T. M. Koper

**Affiliations:** 1 Huygens-Kamerlingh Onnes Laboratory, Leiden University, Niels Bohrweg 2, 2333 CA Leiden, The Netherlands; 2 DESY NanoLab, Deutsches Elektronensynchrotron DESY, Notkestrasse 85, D-22607 Hamburg, Germany; 3 Leiden Institute of Chemistry, Leiden University, Einsteinweg 55, 2333 CC Leiden, The Netherlands

**Keywords:** Electrochemistry, Structure of solids and liquids, Surfaces, interfaces and thin films, Electrochemistry, Surface patterning

## Abstract

In homoepitaxial crystal growth, four basic growth morphologies (idealized growth modes) have been established that describe the deposition of atoms on single crystal surfaces: step-flow, layer-by-layer, mound formation, and random/self-affine growth. Mound formation leads to nano-scale surface patterning. However, the formation of (nano)-islands, patterns, and roughness occurs also during ion bombardment, electrochemical etching and oxidation/reduction cycling. Here we show, in analogy to many particle/anti-particle formalisms in physics, the existence of the dualism between individual adatom and single vacancy growth modes. We predict that all standard adatom growth modes do exist also in their counter, vacancy version. For the particular case of mound formation, we derive the theoretical equations and show the inverse similarity of the solution. We furthermore treat simultaneous growth by adatoms and vacancies, and derive the analytical solution of the growth shape evolution of the mounds. Finally, we present an experimental verification, in which both adatom and vacancy mound formation are active. The theoretically predicted mound shape nicely fits the experimental observation.

## Abstract (web summary)

In homoepitaxial crystal growth, four established modes describe atom deposition on a single crystal surface. Here the authors present a model that shows that, for each adatom growth mode, there exists an analogous but inverse version for vacancy growth. This also applies to combined growth.

## Introduction

In general, one distinguishes several different adatom growth morphologies in crystal growth, which includes not only growth from solution but also e.g. growth under vacuum conditions. Instead of growth morphologies, one often speaks about growth regimes or growth modes, although, strictly speaking, a growth mode is defined by thermodynamics and not kinetics^1^.

**Fig. 1:**
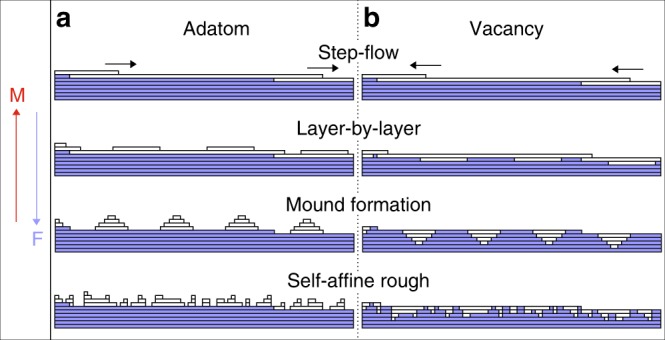
Single-crystal growth regimes: **a** well-established regimes for adatom deposition and **b** newly proposed analogous regimes for vacancy generation during e.g. etching. In which particular regime the surface grows depends on the deposition flux, $F$, divided by the species surface-mobility, $M$, indicated by the arrows

**Fig. 2:**
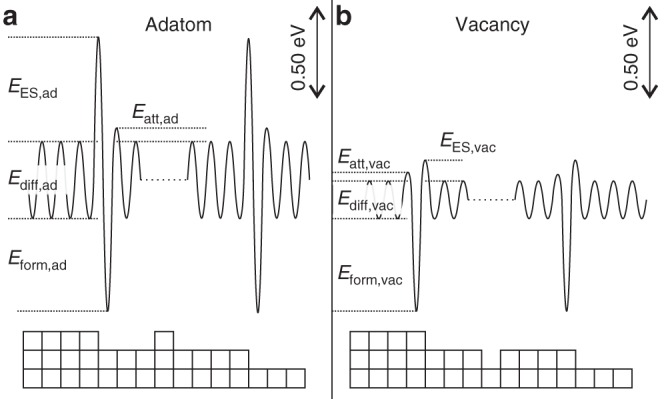
Local potential energy surface for **a** an adatom and **b** a single vacancy on a stepped Cu(100) surface. The energies are drawn to scale based on known values^10–17^

In this paper, we only consider homoepitaxial growth on single crystals, which is often referred to as thin film growth. Under these conditions, four growth morphologies are well established that can be described by four idealized adatom crystal growth modes or regimes^1^, as shown in Fig. 1a. These regimes are characterized and manifested by the diffusion behavior of adatoms on single-crystal surfaces, which is determined by the local potential energy surface for adatoms as shown in Fig. 2a. The existence of the Ehrlich–Schwoebel (ES) barrier, ${E}_{{\mathrm{ES}},ad}$^2,3^, which makes it more difficult for atoms to hop down over a descending step edge than to diffuse over the terrace, is the origin for many different growth phenomena^4–8^.

At low-deposition fluxes, $F$, high-surface mobility, $M$, or small terrace widths between the steps, the rise in surface chemical potential due to the additional adatom pressure on the terrace is too low to overcome the nucleation barrier for islands. Almost all depositing atoms find the ascending step edges during their 2D random walks, where they get incorporated. Only a few atoms overcome the ES barrier and get incorporated in the descending step edge. Effectively, this leads to an uphill adatom flux that stabilizes the terrace widths during growth, resulting in equally spaced step–step distances characteristic for the step-flow growth mode^3,9^.

At slightly higher deposition fluxes, lower mobility, or wider terraces, adatoms meet each other during their random walk on the terraces leading to the nucleation of islands with a typical mean distance that scales with $M/F$^6^. As these islands serve as sinks for newly arriving adatoms, the local adatom pressure and, therefore, the surface chemical potential drops and further island nucleation stops. During the lateral growth of these islands, the probability for nucleation events on top of them increases. However, as the mean distance between the originally nucleated islands is determined by the mean-free diffusion path of the arriving adatoms, most atoms overcome the ES barrier and get incorporated into the rim of the island. As a consequence, (almost) no second-layer islands nucleate before the original layer is (almost) completely filled. This leads to the layer-by-layer growth mode, Fig. 1a.

At even higher deposition fluxes or lower mobility, mound formation occurs: island nucleation continuously occurs on top of growing (previously nucleated) islands. With a significant ES barrier, the steps of the mounds grow mainly by the attachment of atoms that land on the lower terrace of a particular step edge. Without interlayer mass transport (very high or infinite ES barrier), the step growth speed becomes proportional to the lower terrace width, and a unique set of differential equations can be formulated that describes the evolution of the mound shape^9^. An important ingredient in the mound evolution is the decrease of the growth speed of the lowest step. The reason for this is that its lower terrace decreases towards zero width by either getting closer to the lowest step of the neighboring mound (single-crystal growth^18^) or by approaching a grain boundary (polycrystal growth^19^). As a consequence, steps that are located lower in the mound structure, grow slower: steps are piling up, additional roughness is created, and the mounds are stabilized. This “race” between the steps, in which the upper, faster step is never capable of catching up with the lower, slower one, is also known as the *Zeno Effect* (by analogy with Zeno’s paradox about the race between Achilles and the tortoise)^20^. With these boundary conditions and a scaling to the base width of the mound (normalized mound radius), the differential equations can be solved analytically: the mound shape follows a Poisson distribution^1,9^. Based on the same principles, mound formation occurs also with a finite ES barrier that allows for (some) interlayer mass transport. The shape, however, differs significantly from a Poisson distribution, showing mounds with flat tops^1,9^.

At very high fluxes or low mobility, the adatoms have no time to (extensively) probe the local potential energy surface to find a (local) minimum. Following the solid-on-solid approach, in which overhangs are forbidden, a self-affine, rough growth front is created. Its evolution can often be described by scaling laws following particular universality classes^7^.

It is interesting to notice that mounds can be also formed during ion beam etching, which in addition often leads to pattern formation producing self-organized ripple-like, dot-like, or hole-like structures. There are still many open fundamental questions on the underlying atomic processes that are responsible for this pattern formation^21–26^. As vacancy creation is clearly one of the most prominent atomic processes during ion beam etching, it is, therefore, important to also consider vacancy growth morphologies.

## Results

### Prediction: vacancy growth morphologies

All phenomena reviewed above rely on atom deposition and the ratio between surface diffusion on terraces and over step edges, as illustrated in Fig. 2a. Naturally, when considering etching, the question arises if analogous, antiparticle growth models for vacancies exist, as sketched in Fig. 1b. The requirements for equivalence are the random generation (“deposition”) of vacancies in combination with the existence of a vacancy ES barrier ${E}_{{\mathrm{ES,vac}}}$. It is well known that vacancies can be created by noble gas ion bombardment (sputtering), electrochemical etching, and chemical reactions (like oxidation and/or reduction). Probably less known is the existence of the ES barrier for vacancies, as shown for Cu(100) in Fig. 2b based on theoretical and experimental values^10–17^: when a vacancy approaches an ascending step edge, the above atom needs to pass over a less favorable transition state to hop into the lower vacancy, thereby, effectively incorporating the vacancy into the upper part of the step. When comparing the energy landscape for adatoms and vacancies in Fig. 2, one notices similar formation energies for both species. This means that the equilibrium densities of vacancies and adatoms in/on the terrace are similar at any given temperature. However, based on the results in ref. ^10^ vacancies “race” through the terrace, due to their much lower diffusion barrier ${E}_{{\mathrm{diff,vac}}}$. On the other hand, theory has suggested that the mobility of adatoms and vacancies is similar^17^. The existence of single vacancies as well as their diffusion within the first layer has been experimentally proven with an STM already in 1997 by the use of so-called tracer atoms^27^. In addition, vacancy mound formation on one single mound has been observed recently during sublimation of Si(111)^28^. With this knowledge, one clearly expects that all four adatom growth modes are similarly realized also for vacancies, as depicted in Fig. 1b.

**Fig. 3:**
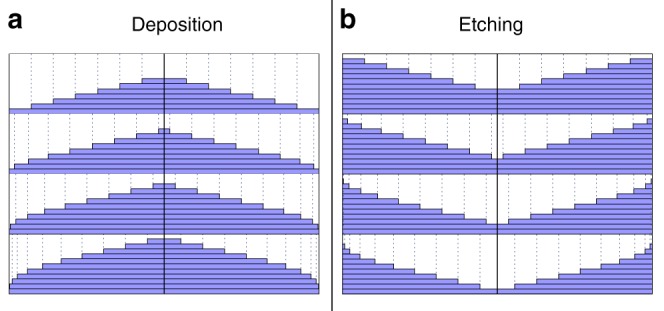
Zeno Effect: Shape evolution of the mounds formed during deposition (**a**) and etching (**b**), due to the Zeno Effect^18–20^

Considering the third growth mode, one realizes that the final surface contour looks rather similar for both adatom deposition and vacancy creation: in both cases (atom) mounds are formed, see Fig. 1. However, upon closer inspection it becomes clear that the Zeno Effect is inverted: during atom deposition, the steps are piling up at the bottom of the mounds, whereas they are getting closer together at the top of the mounds during vacancy creation; compare Fig. 3a, b.

**Fig. 4:**
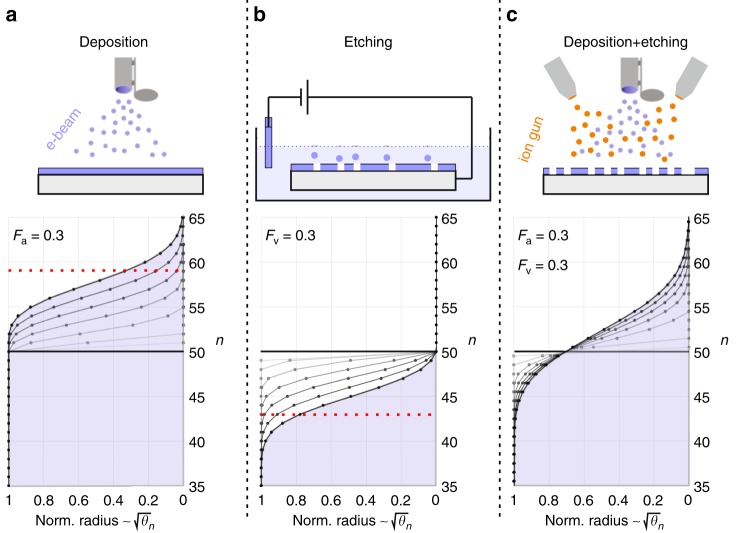
Three scenarios for mound formation: **a** The stochastic deposition of atoms (or molecules) leads to mound formation during growth. **b** An inverted mound shape is obtained during etching (“deposition” of vacancies) that occurs e.g. during electrochemical etching, sputtering of the surface or a chemical reaction. **c** The combined “deposition” of adatoms and vacancies, as occurs e.g. in ion beam assisted deposition (IBAD), leads to an accelerated evolution of the mound shape, which is depicted in the lower panels. The evolution is calculated via the theory described in the text, in which the radius of the mound (base) is normalized to one. The *Y*-axis represents the number, $n$, of the atomic layer, ${\theta }_{n}$, in the mound. To enable a comparison between the different scenarios, we included an offset to the original flat surface leading to the starting condition: ${\theta }_{n}({t}_{0})\,=\,1\ \forall \ n\le 50\ \wedge \ {\theta }_{n}({t}_{0})\,=\,0\ \forall \ n\; > \; 50$. Due to the nucleation barrier for the topmost island and a large, but finite ES barrier, the real mound shape changes slightly and shows flat tops indicated by the red dashed line in **a**, see ref. ^1^ for more details. Similarly, the existence of an analogous vacancy island nucleation barrier cuts the vacancy island mound shape, see red dashed line in **b**

Finally, one should distinguish three different scenarios for mound formation, as sketched in Fig. 4: atom deposition, vacancy creation, and a combination of both. The latter is realized not only during ion beam assisted deposition and sputter deposition, but also during ion bombardment (sputter cleaning) and surface oxidation/reduction cycles under both gas-phase^29^ and electrochemical conditions^30^. During these processes surface adatoms, adatom islands, and vacancies are created by either high energy ion impacts or oxidative roughening.

### Theoretical description for mound formation

In the following, we develop the theoretical description for mound formation considering simultaneous adatom and vacancy deposition. Assuming no interlayer mass transport (infinitely high ES barrier), it is possible, analogous to the theory by Michely and Krug^1^, to formulate a generalized analytical equation for the mound shape. If ${\theta }_{n}$ describes the normalized coverage of layer $n$ ($0\le {\theta }_{n}\le 1$), the rate of coverage change of layer $n$ can be expressed by

1 $$\frac{d{\theta }_{n}}{dt}\,=\,\Omega {F}_{a}({\theta }_{n-1}\,-\,{\theta }_{n})\,-\,\Omega {F}_{v}({\theta }_{n}\,-\,{\theta }_{n+1}),$$

in which $\Omega$ is the atomic volume and ${F}_{a}$ and ${F}_{v}$ the fluxes of “arriving” atoms and vacancies, respectively. In the case of deposition only, ${F}_{v}\,=\,0$, it has been shown^1,9,31,32^ that

2 $${\theta }_{n}\,=\,1\,-\,{e}^{-{\Theta }_{a}}\sum _{k=0}^{n-1}\frac{{\Theta }_{a}^{k}}{k!}$$

follows a Poisson distribution with the total deposited amount ${\Theta }_{a}\,=\,{\sum }_{n}{\theta }_{n}\,=\,\Omega {F}_{a}t$, in which *t* is the time. Note that in reality a nucleation barrier exists for the topmost adatom island that leads to flat mound tops (cut Poisson shape), as indicated with the red dashed line in Fig. 4a. The magnitude of the ES barrier affects the size of the mound tops: the lower the barrier the larger the tops. Mounds that are formed with a finite ES barrier that allows for interlayer mass transport differ significantly from the Poisson shape. The larger the deviation the lower the ES barrier is. These shapes are best described by mesa-shaped mounds with large flat tops separated by deep crevices. For minor interlayer mass transport the shape can be approximated by a cut Poisson shape. For more details see ref. ^1^.

In the case of etching only, ${F}_{a}\,=\,0$, one obtains exactly the same shape (Poisson distribution) for the etching holes into the crystal as the mound shapes that grow on top during deposition only. This can be derived by inverting the counting direction of layers $n$: into the crystal instead of on top. If one considers the shape of the remaining mounds on the surface (and not the holes), any shape asymmetry is mirrored in the height direction, as sketched in Fig. 3a, b. This is only important for the early stages of the evolution, as the Poisson distribution becomes (almost) symmetric for large $\Theta$ in the later stages. In the case of simultaneous etching and deposition, the evolving mound shape equals the shape of a Poisson distribution with a higher effective flux ${F}_{a}\,+\,{F}_{v}$ (variance), whereas the height (mean value) is determined by the flux difference $| {F}_{a}\,-\,{F}_{v}|$. Mathematically this is described by the subtraction of two Poisson distributions, called Skellam distribution^33^, as long as the “deposition” events are two independent stochastic processes. Its properties and applications have been discussed in ref. ^34^ and the general solution to Eq. 1 is, therefore, given by

3 $${\theta }_{n}\,=\,1\,-\,{e}^{-({\Theta }_{a}\,+\,{\Theta }_{v})}\sum _{k=0}^{n-1}{\left(\frac{{\Theta }_{a}}{{\Theta }_{v}}\right)}^{k/2}{I}_{k}(2\sqrt{{\Theta }_{a}{\Theta }_{v}}),$$

in which $\Theta \,=\,{\sum }_{n}{\theta }_{n}\,=\,{\Theta }_{a}\,-\,{\Theta }_{v}\,=\,\Omega {F}_{a}t\,-\,\Omega {F}_{v}t$ and ${I}_{k}(x)$ are the *modified Bessel function of the first kind*^34^. Figure 4 shows the evolution of the surface profile for all three types of mound formation. For clarity, only the left halves of the radially symmetric mound shapes are shown. Note that the size of the layers within the mounds depends on the number of the atomic layer in the mound.

### Experimental verification

**Fig. 5:**
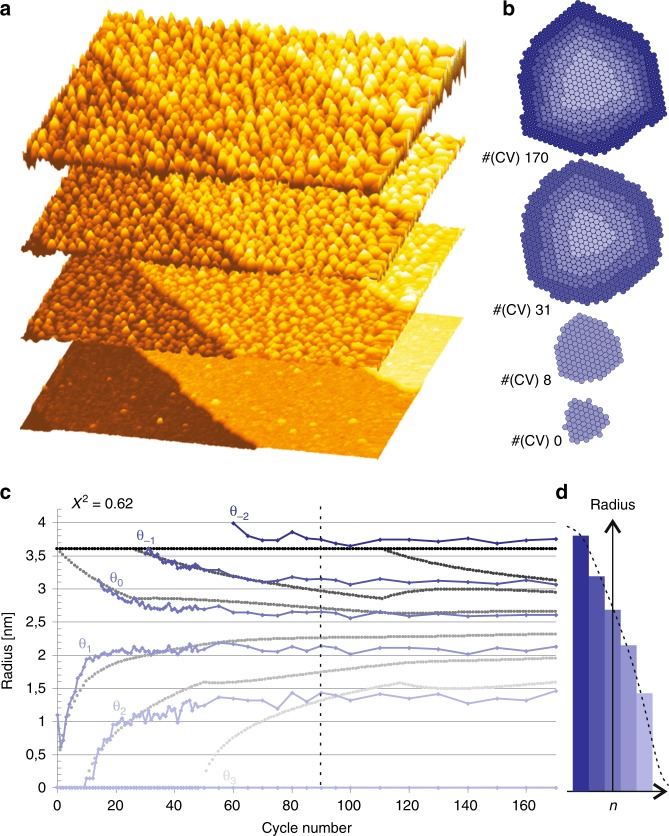
Mound formation during combined vacancy and adatom creation: **a** nanoislands are formed during the electrochemical oxidation/reduction cycling on Pt(111)^30^; **b** average, atomic island shape extracted from **a**, see ref. ^35^; **c** the evolution/growth of the individual atomic layers within the mounds, provided as radii in the graph, are obtained from the average island shape per cycle that typically takes into account a minimum of 400 islands^35^. The layers are indicated from light to dark blue. All corresponding gray lines are obtained by one single fit of the theory with only four fitting parameters, see text. **d** The island shape in layer filling representation at the moment of the dashed line in **c**

We illustrate mound formation by combined adatom and vacancy deposition with an electrochemical system: nanoislands (mounds) nucleate and grow on a well-defined Pt(111) single-crystal surface upon repetitive oxidation/reduction cycling^30^. Figure 5a shows four selected microscopy images during the mound evolution in which 170 roughening cycles were applied. Figure 5b shows the corresponding atomic configurations of the average mound shape^35^. This system fulfills the conditions for simultaneous etching and deposition. During the oxidation sweep, atoms are pushed out of the terraces^36^. As not all atoms find their way back to their specific sites of origin, vacancies are created during the reduction sweep^30,37^. Effectively, atoms are “deposited” on the terraces, while vacancies are created simultaneously. It is known that small amounts of platinum are lost in the electrolyte, but this is less than one monolayer for the complete duration of our experiment covering 170 cycles^38^. Therefore, we approximate the system with mass conservation leading to mound formation with ${F}_{a}\,=\,{F}_{v}$.

Figure 5c shows the experimentally determined island filling/radii (blue data) of the first five open atomic layers in the developing mounds as a function of the oxidation–reduction cycle (ORC) number. The size of a layer within a mound depends on its atomic layer, whereas the size of the mound is constant after the initial growth to its maximum size. Please note that these data are extracted from an *average island growth shape* per cycle (see “Methods” section), like the ones shown in Fig. 5b, which typically takes into account a minimum of 400 islands. The radii are, therefore, a good representation at a given time during the statistical evolution of the mound shape. With respect to ref. ^35^, the methodology to fit atomic structures consisted of one additional step. Here, we take into account the height distributions of each of the images to determine their relative height offset. This enables us to keep track of the original terrace layer and distinguish the different vacancy and adatom layers. The full series of fit results is provided as Supplementary Movie 1. Upon closer inspection of the fit data in Fig. 5c, one realizes that (1) the island radii show an asymptotic behavior with a center at around 2.4 nm; (2) the distances between the individual layers/steps steadily decrease during the evolution/growth of the mounds; (3) the growth of both a next higher and a next lower layer are significantly delayed, note e.g. the delay of ten cycles between ${\theta }_{1}$ and ${\theta }_{2}$; (4) the rise (growth speed) of the layers follows a characteristic evolution determined by the flux of atoms and vacancies.

All observations are in good agreement with the general theory described above. The nucleation of the islands/mounds in combination with the Zeno Effect is responsible for (1) and (2). Eventually, this leads to a self-organization on the surface with a hexagonal pattern and a certain developed length scale between the islands^6^. The delay of the next layers (3) is due to the existence of a nucleation barrier for the stable nucleus. An individual atom is mobile enough to find its way down over the step edges. The mobility of a dimer is expected to be still significantly high. A trimer, on the other hand, forms a compact cluster with a large diffusion barrier and is expected to serve as a stable nucleus. In general, *magic clusters* with 1, 3, 7, 19, etc. atoms are rather stable^39^. Notice that the delayed appearances of ${\theta }_{-1}$ and ${\theta }_{-2}$ imply the existence of a nucleation barrier for vacancy islands as well.

Due to the existence of the nucleation barriers, the evolution cannot be analytically solved with Eq. 3. Instead we use a numerical solution with rate equations and finite time steps that describes Eq. 1 and takes into account additionally nucleation barriers. These combined differential equations have only four independent free variables to fit the complete data-set with all five layers and 330 individual data points: the *maximum mound radius* that determines the distance between the islands and, thereby, the maximum value a layer can have. Larger values rescale the *Y*-axis, but leave the relation between the different layers unaltered; the *percentage of atoms* that is pushed out during the oxidation determines the growth speed $F\,=\,{F}_{a}\,=\,{F}_{v}$ and, therefore, the characteristic slope of the radii of the individual layers in time; the *critical nucleation radius for adatom and vacancy islands* determines the delay between the individual layers as well as the size of the top and the bottom, flat terrace of the mound. All four parameters clearly have their own specific influence on the appearance of the fit data in Fig. 5c; see Supplementary Note 1 for more detailed information on the specific influences of these parameters.

All gray lines are simultaneously obtained with only one single fit by the theory. With a chi-square of only *X*^2^ = 0.62, we obtain the following results: maximum mound radius = 3.6 nm, percentage of atoms = 0.0245 ML, and critical nucleation radius adatoms = 0.21 nm and vacancies = 0.41 nm. The critical radii imply that the critical nuclei are given by a dimer, trimer, or slightly larger size island. Inspecting the fit further, one notes that an adatom layer, ${\theta }_{3}$, is predicted by the theory, which is not seen experimentally. The reason might be that we lose up to one monolayer of platinum in our experiment. Furthermore, one sees experimental data points, ${\theta }_{-2}$, beyond the 3.6 nm of our fit. Note that the Poisson distribution is symmetric and that this symmetry lies around 2.4 nm in our case. If we choose a larger radius, all lines shift up leading to a better fit for ${\theta }_{-2}$, but a significantly worse fit with the symmetry enveloped by ${\theta }_{1}$ and ${\theta }_{0}$. This deviation is probably due to tip convolution effects. Anyhow, one should realize that we obtain a reasonable fit with only four parameters describing the overall evolution and, therefore, we consider this as a suitable verification of the above described model.

In conclusion, in this paper, we predict and describe a complete set of analogous, homoepitaxial growth modes that are all based on the “deposition” of single, individual vacancies or a combination of vacancies and adatoms. For the particular case of mound formation due to vacancy deposition only, we show theoretically that the evolving mound structure can be described by an inverted Poisson shape, similar to the evolving mound shapes during pure atom deposition. Considering the simultaneous deposition of adatoms and vacancies, we derive and solve the theoretical equations leading to an analytical description of the growth shape evolution: the shape is determined by a Skellam distribution, which describes the subtraction of two Poisson distributions. We verify our theoretical predictions for the simultaneous growth of adatoms and vacancies on the example of the nanoisland/mound formation during oxidation/reduction cycling of Pt(111) in electrochemical environment: the complete evolution of the first five mound layers over all 170 cycles can be fit satisfactorily by only four variables (island size, flux, and critical nucleation sizes) when applying the theory introduced here. Next to the general insight, we predict that the existence of vacancy mediated mound formation, as described in this article, will also shed new light on both the still open fundamental questions and the underlying atomic processes responsible for the pattern formation during ion beam etching that produce self-organized ripple-like, dot-like, or hole-like structures^21–26^.

## Methods

### Experimental details

All experiments were carried out with a home-built EC-STM set-up described previously^40–42^. It has been shown that this set-up delivers high-resolution images, also on relatively rough surfaces, while still operating with significant scanning speeds. To allow for long-term experiments, for maintaining accurate control over the potentials, and for a high degree of cleanliness, we developed a new EC-STM cell with a reversible hydrogen reference electrode (RHE) that is compatible with the STM. A coiled platinum wire is used as counter electrode.

The earlier performed experiment contains 170 ORCs between 0.06 and 1.35 V^30,35^. Although our STM is capable of measuring in operando during a cyclic voltammogram (CV), our aim was to record high-quality snapshots, in which the complete imaged surface has transformed in the same way. Therefore, all images are acquired in the double-layer region at constant tip and sample potential (${U}_{t}$ = 0.45 V and ${U}_{s}$ = 0.4 V, respectively).

Before starting the experiment, the surface quality and cleanliness were checked by (large scale) STM imaging and cyclic voltammetry. Under these conditions, the sample potential (${U}_{s}$) is not scanned above 0.85 V versus RHE to prevent any change in the surface structure. Directly before and after each ORC, we again record CVs up to 0.85 V to monitor the appearance of possible contaminants in between the oxidation experiments. All CVs are performed with the tip fully retracted (1 µm away) and at a fixed potential (${U}_{t}$ = 0.45 V) to minimize the number of tip switches. Performing the ORCs with a retracted tip also prevents changes in the local sample potential due to the extremely small distance and large potential difference between the two electrodes. Such local potential changes would imply that the imaged area is no longer representative for the entire electrode surface. After each oxidation–reduction sequence (single ORCs for cycle 1–50, five ORCs for cycle 55–100, and ten ORCs for cycle 110–170) an STM image is acquired. During imaging, the sample and tip potentials are fixed at ${U}_{s}$ = 0.4 V and ${U}_{t}$ = 0.45 V, respectively. This approach leads to a total data acquisition time of 17 h.

### Determination of Island Radii

The radii data used in the Experimental Verification are extracted from an earlier experiment described in ref. ^35^. In general, all islands of one image are averaged to obtain the *average island growth shape* per cycle. As this typically means an average of 400 islands, the islands radii are a good representation at a given time during the statistical evolution of the mound shape. To account for the finite tip size and its inherent convolution, we first calculated tip deconvolutions to the STM images assuming a sphere with different, increasing radii. Above a radius of 1.5 nm features occur in the calculated images that are smaller than one atom. On top of this, we also accounted for a significant radial asymmetry of the tip, which is manifested in the asymmetry of the average island growth shape. From repeated similar experiments with other tips, we know that this asymmetry is indeed a tip effect and that the formed islands exhibit a threefold symmetry. If one would correct the data for both the spherical convolution and the tip asymmetry, one would overestimate the correction. The tip asymmetry seems to have the largest influence on our analysis, which is why we employ a threefold island symmetry as boundary condition in the fitting procedure to correct for it. As any tip size/shape will always lead to an overestimation of the island shape, we argue that the minimum height in all directions of the threefold symmetry describes the real average island shape most accurately. This approach also minimizes the general tip convolution effect, but no additional corrections are applied to further reduce it. To obtain the atomic ball models, the inner contour of the corrected *average island growth shape* is then fitted further with an atomic scale model. More details on the deconvolution and fit procedure are provided in ref. ^35^. Furthermore, to account also for vacancy islands, we here keep track of the height distributions and, in particular, also the relative height offsets between the images. In comparison with ref. ^35^, this enables us to keep track of the original terrace layer and to distinguish between the different vacancy and adatom layers. The new, full series of fit results with vacancy islands is provided as Supplementary Movie 1.

## Supplementary information

Supplementary Information

Description of Additional Supplementary Files

Supplementary Movie 1

## Data Availability

The original data sets analyzed during the current study are not publicly available, as they are obtained with home-written control software that is not publicly available. No other existing software is capable of reading our original microscopy data. However, an export into a standard format could be generated by the corresponding author upon reasonable request. In addition, all uncompressed microscopy images that support the findings of this study can be downloaded in form of a movie via the Supplementary Material of ref. ^30^. All extracted island shapes determined in this study can be downloaded in form of a Supplementary Movie 1.

## Code availability

All data processing is performed with home-written routines using WaveMetrics Igor Pro software. Details are provided in the Supplementary Information of ref. ^35^. The theoretical fit to the experimental data is performed with MS Excel. Both the home-written routines in WaveMetrics Igor Pro as well as the MS Excel file are available upon reasonable request to the corresponding author.
