# Supplementary material for: The dualism between adatom- and vacancy-based single crystal growth models
Source: Nat Commun. 2019 Nov 20;10:5233. doi: 10.1038/s41467-019-13188-0 (PMC6868172; doi:10.1038/s41467-019-13188-0)
Supplement: Supplementary file 1 — Supplementary Information [file 41467_2019_13188_MOESM1_ESM.pdf]

Supplementary Information on

"The Dualism between Adatom- and Vacancy-Based Single Crystal Growth Models"

by

Marcel J. Rost [1], Leon Jacobse [2,3], and Marc T.M. Koper [3]

1) Huygens-Kamerlingh Onnes Laboratory, Leiden University, Niels Bohrweg 2, 2333 CA Leiden, The Netherlands
2) DESY NanoLab, Deutsches Elektronensynchrotron DESY, Notkestrasse 85, D-22607 Hamburg, Germany
3) Leiden Institute of Chemistry, Leiden University, Einsteinweg 55, 2333 CC Leiden, The Netherlands

Supplementary Note 1:

Here we discuss the specific influences of the fit parameters on the figures.

The theoretical fit shown in Fig. 4c in the main text depends on only four fit parameters: the *maximum mound radius*, the *percentage of atoms/vacancies* that is created during one oxidation/reduction cycle (flux), and the *critical nucleation radius for both adatom and vacancy islands*. As each of these four fit parameters has its very own, specific influence on the appearance of the fit curves, there is only one set of parameters that fits the experimental data sufficiently well. In other words, there are no local minima for the quality of the fit results (X^2). In the following we highlight the specific influences of the four parameters with examples.

The *maximum mound radius* is determined by the distance between the islands/mounds. It describes the size of the unit cell and, thereby, the maximum size a mound can have. This parameter scales with respect to the Y-axis. Supplementary Fig. 1 shows the evolution of the layers of the mounds for a *maximum mound radius* of 3.6 nm (a) and 7.2 nm (b). Note that the Y-axis in (b) is also scaled with a factor of two! The curves, and therefore the evolution, are exactly the same on these scales. This means that the *maximum mound radius* predominately shifts all curves up and down (along the Y-axis).

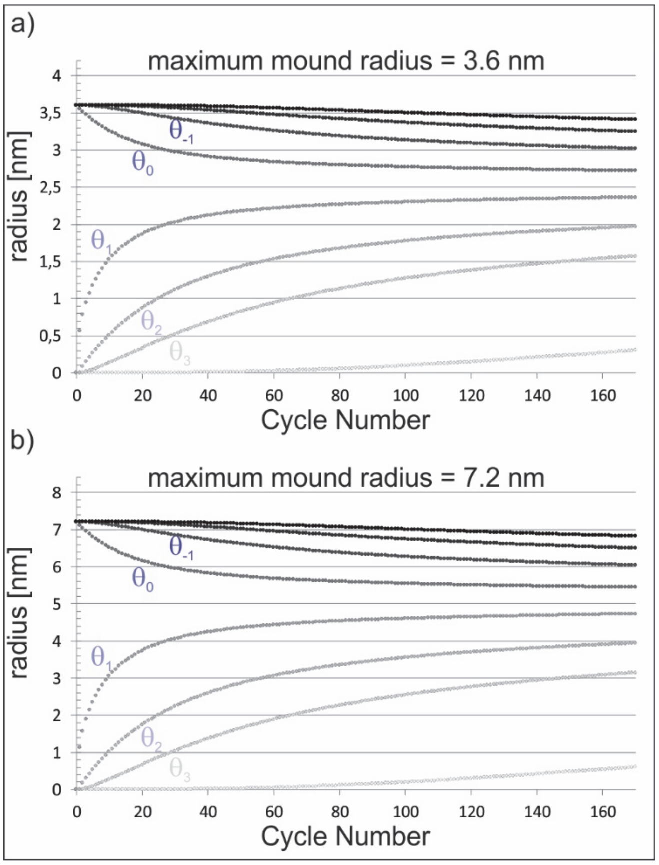

Supplementary Fig. 1: *The evolution of the layers of the mounds is calculated for different maximum mound radii: 3.6 nm in (a) and 7.2 nm in (b). Note that the Y-axis in (b) is also scaled with a factor two. As the curves are exactly the same on these scales, the maximum mound radius shifts all curves up and down along the Y-axis. The percentage (flux) of atoms and vacancies that are created is 0.0245 ML (monolayer) and we did not include a nucleation barrier for adatoms or vacancies.*

The *percentage of atoms/vacancies* that is created during one oxidation/reduction cycle describes the flux of the ‘deposition’. The larger the flux is the faster is the evolution of the mounds during their growth. Supplementary Fig. 2 shows the evolution of the layers in the mounds for a flux of 0.0245 ML (monolayer) (a) and 0.049 ML (b), respectively. The *maximum mound radius* is 3.6 nm in both cases. Note the accelerated evolution/growth of the mounds for the higher flux. The curves of the individual

layers are pushed much quicker together and there is a higher curvature of the individual curves in the early stages of the evolution. The *percentage of atoms/vacancies* determines the speed of the evolution!

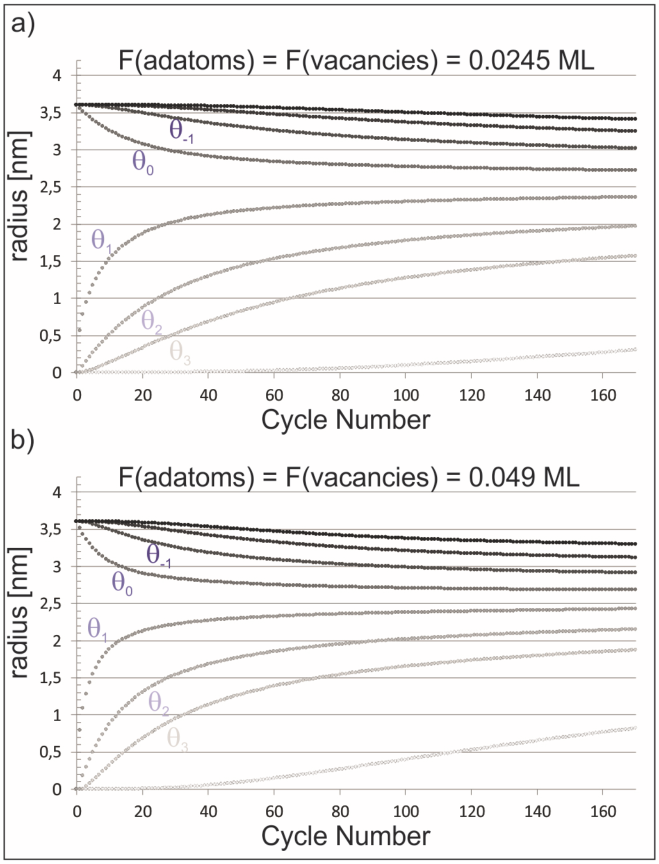

Supplementary Fig. 2: *The evolution of the layers of the mounds is calculated for different adatom/vacancy fluxes, 0.0245 ML in (a) and 0.049 ML in (b). Note the accelerated growth for higher fluxes: the curves are pushed together and there is a higher curvature of the individual curves in the early stages.* The percentage of atoms/vacancies determines the speed of the evolution! *The maximum mound radius is 3.6 nm and we did not include a nucleation barrier for adatoms or vacancies.*

Until now we did not include a nucleation barrier for adatom or vacancy islands. As a consequence all adatom islands and all vacancy islands of the mounds start to grow right from the beginning (1st

oxidation/reduction cycle). This situation is sketched one more time in Supplementary Fig. 3a, which is

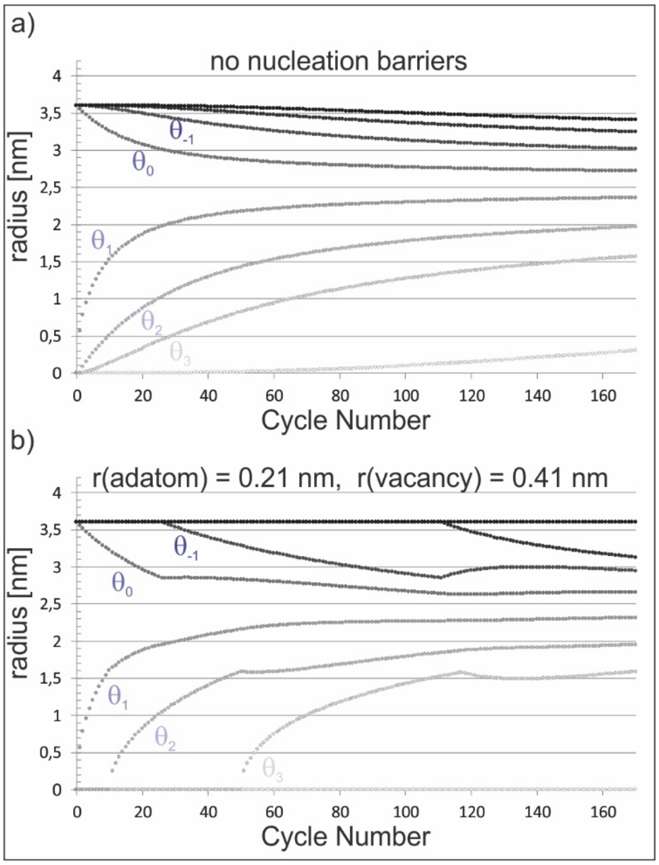

Supplementary Fig. 3: *The evolution of the layers of the mounds is calculated for no nucleation barriers in (a) and a critical nucleation radius for adatom islands of 0.21 nm and vacancy islands of 0.41 nm in (b). Note the significant delay of the growth of the next layers: the delay is larger for vacancy islands due to the larger critical nucleation radius. The flux of adatoms/vacancies is 0.0245 ML and the maximum mound radius is 3.6 nm.*

calculated for a *maximum mound radius* of 3.6 nm, a flux (*percentage of atoms/vacancies)* of 0.0245 ML and no nucleation barriers. In reality, however, we see a delay of the growth of the next layer for both adatom as well as vacancy islands. This means that we have to include critical nucleation radii for adatom and vacancy islands, shown in Fig. Supp. 3b. Note the significant delays of the growth of the

next layers: they are larger for the vacancy islands, as the critical radius for vacancies is assumed to be 0.41 nm, whilst it is only 0.21 nm for adatom islands. The larger a critical radius the larger is the corresponding delay.

Summarizing the individual influences of the four parameters, one can state that the *maximum mound radius* determines the total size of the mounds and shifts, therefore, all curves up and down along the Y-axis. Scaling applies here. The *percentage of atoms/vacancies* determines the speed of the evolution: with higher flux the curves are pushed together and there is a higher curvature of the individual curves in the early stages of the growth. Finally the *critical nucleation radius for both adatom and vacancy islands* determines the delay of the growth of the next layer, respectively.

These specific influences are independent of each other, which means that a final fit of the experimental data is straight forward. We varied the different parameters and determined the best fit by the lowest X^2 value.
