# Supplementary material for: The dualism between adatom- and vacancy-based single crystal growth models
Source: Nat Commun. 2019 Nov 20;10:5233. doi: 10.1038/s41467-019-13188-0 (PMC6868172; doi:10.1038/s41467-019-13188-0)
Supplement: Supplementary file 2 — Description of Additional Supplementary Files [file 41467_2019_13188_MOESM2_ESM.pdf]

Description of Additional Supplementary Files

File Name: Supplementary Movie 1
Description: The movie shows the atomic ball model fits to the average growth island shape as a function of the number of oxidation/reduction cycle (ORC). It contains one fit/image for each single ORC for cycle 1 - 50, one fit/image per five ORCs for cycle 55 – 100, and one fit/image per every 10 ORCs for cycle 110 – 170. For more details on the fit see the section "Methods" in the main text.
